# RNA-Seq profiling of circular RNAs in human laryngeal squamous cell carcinomas

**DOI:** 10.1186/s12943-018-0833-x

**Published:** 2018-05-01

**Authors:** Cheng Lu, Xi Shi, Amanda Y. Wang, Yuan Tao, Zhenxiao Wang, Chaoping Huang, Yuehua Qiao, Hongyi Hu, Liangfa Liu

**Affiliations:** 1 Department of Otolaryngology, Head Neck Surgery, Beijing Friendship Hospital, Capital Medical University, 95th Yong’an Road, Xicheng District, Beijing, 100050 China; 2 The Institute of Audiology and Speech Science of Xuzhou Medical Collage, Xuzhou, 221004 China; 3 The renal and Metabolic Division, The George Institute for Global Health, UNSW, Australia; Faculty of Medicine & Health Sciences, Macquarie University, Sydney, NSW 2109 Australia; 4 Department of Otolaryngology, Peking University Shenzhen Hospital, Shenzhen, 518036 China

**Keywords:** Circular RNAs (circRNAs), RNA-sequencing (RNA-Seq), Laryngeal squamous cell carcinomas (LSCC)

## Abstract

Abnormal expression of non-coding circular RNAs (circRNAs) have been reported in many types of tumors. circRNA have been suggested to be an ideal candidate biomarker for diagnostic and therapeutic implications in cancers. The aim of this study was to assess the circRNA expression profile of laryngeal squamous cell carcinomas (LSCC). The biopsy samples from patients with LSCC were obtained intra-operatively. The circRNA expression was performed using secondary sequencing. Among 10 patients with LSCC, 2 were well differentiated, 3 were moderately differentiated and 5 were adjunctive samples with normal and LSCC tissues. A total of 21,444 distinct circRNA candidates were detected. Among them, we defined the statistical criteria for selecting aberrant-expressed circRNA using a q-value of < 0.001 with a fold change of > 2.0 or < 0.5. A total of 29 circRNA were upregulated and 19 circRNA were downregulated significantly in the LSCC tissues. The intersection of these dysregulated circRNAs of normal-well differentiated set and normal-moderately differentiated set was then assessed to narrow the upregulated and downregulated circRNAs down to 18 and 5 respectively. Furthermore, an association of the circRNA-miRNA-mRNA was investigated, showing that 20 dysregulated circRNA successfully predicted an interaction with several cancer-related miRNAs. Finally, a further KEGG analysis showed that PPAR, Axon guidance, Wnt and Cell cycle signaling pathway were key putative pathways in the process of LSCC. hsa_circ:chr20:31876585–31,897,648 was found to be able to differentiate most of LSCC from the matching normal tissues. This observational study demonstrated dysregulation of circRNA in LSCC, which may have an impact on development of potential biomarkers in this disease. Validation of down-regulation of hsa_circ:chr20:31876585–31,897,648 in LSCC compared to each adjunctive tissue by Q-RT-PCR, indicating that hsa_circ:chr20:31876585–31,897,648 may be a novel promising tumor suppresser in LSCC.

**Electronic supplementary material:**

The online version of this article (10.1186/s12943-018-0833-x) contains supplementary material, which is available to authorized users.

## Main text

Laryngeal squamous cell carcinomas (LSCC) is a malignant carcinoma derived from the epithelial tissue of laryngeal mucosa. It is an aggressive head and neck neoplasm, which makes up approximately 2.4% in all emerging malignant tumors worldwide every year [1]. Moreover, there is an increasing incidence of LSCC in recent years. Multiply factors such as smoking and excessive alcohol consumption are contributing to the development of LSCC. Although recent research on LSCC have made some remarkable achievements, the etiology and pathogenesis underlying this disease development remains unclear. Over the last decade, despite of increasing numbers of research with a focus on identification of biomarkers to improve clinical outcomes of LSCC, LSCC is still associated with high morbidity and mortality. In particular, its prognosis remains very poor for patients with metastatic diseases, with a 5-year mortality rate of more than 50% [2]. Therefore, better understanding of biomarkers associated with LSCC can play an important role in disease stratification and prognostication. It can also potentially inform the development of novel targeted therapies.

In recent years, identification of biomarkers in certain types of tumors have led to development of targeted therapies and improved clinical outcomes for biomarkers derived subsets of patients. To date, the dysregulated expression of non-coding RNAs including lncRNA, microRNAs have been closely associated with pathogenesis of some human cancers. Several non-coding RNA [3] have shown to be significantly changed in different sorts of cancers [4]. Circular RNAs (circRNAs) are one of non-coding RNA discovered lately. They are produced by linking the 3′ and 5′ ends via the covalent bonds to form a closed loop, circRNAs. Due to this closed structure, circRNAs have been demonstrated to have a high level of stability and resistance to RNA degradation pathways [5]. Therefore, they may be more promising and technically suitable biomarkers for cancers [6]. Recent literatures have shown dysregulated expression of several circRNAs in human cancers [7]. Moreover, resent studies have confirmed that the expression of circular RNAs is different between the LSCC sample and its adjacent tissues via microarray detection [8].

Despite several potential circRNA biomarkers have been detected in LSCC, due to the limitation of using microarray technology that could only focus on the known candidate circRNAs preset on the chip, a large number of novel promising circRNAs in LSCC beyond this microarray analysis are waiting for further screening. Moreover, to our knowledge, there are no studies to date have yet profiled the relationship of the circRNA-miRNA-mRNA expression in human LSCC in details. Therefore, the aim of this study was to profile the LSCC related dysregulated circRNA via secondary sequencing technology to identify novel circRNA biomarkers. Meanwhile, the relationship of these circRNA and their potential binding miRNA-mRNA were further analyzed to improve our understanding of the pathogenesis of this disease.

## Collection and grouping of the LSCC samples based on the pathological classification

Specimens were collected from patients with laryngeal cancer who underwent laryngectomy at the Department of Otolaryngology, Head Neck Surgery, Beijing Friendship Hospital affiliated to Capital Medical University, China between August 2016 and December 2017. Secondary sequencing was used to test 10 matched samples of the LSCC tissues and corresponding adjacent non-neoplastic tissues. Ten matched cancerous and noncancerous tissues were tested by real-time qPCR. The study was approved by the Ethics Committee of Beijing friendship hospital. The patients who had received any cancer therapy before admission were excluded from this study. After immediate snap-frozen in liquid nitrogen post-resection, these specimens were stored at − 80 °C. Histopathological examination of the laryngeal tissue specimens was independently carried out by two pathologists. After histopathological vetting, a total of ten laryngeal samples–consisting of five LSCC (which included three moderately differentiated and two well differentiated specimens), and five matching contralateral normal tissue samples were finally included in the study.

## RNA isolation and next generation RNA sequencing analysis

RNA isolation was performed according to standardized protocols in an RNA-dedicated work area with an access to RNase/DNase-free water and RNase-free labware. In brief, total RNA was extracted from the samples with TRIzol reagent (Invitrogen, Carlsbad, CA, USA) according to the kit’s instructions. The resulting RNA pellet was washed twice in 75% ethanol (1 ml), then air-dried, and re-suspended in RNase/DNase-free water (20 μl). After that, the Turbo DNase Kit (Ambion) was applied to the RNA solution to degrade any remaining DNA. The RNA was subsequently purified and concentrated with an RNeasy MinElute Cleanup kit (Qiagen, Valencia, CA, USA). The total RNA concentrations in the sample tissues and quality of each sample were then assessed with a NanoDrop ND1000 spectrophotometer (NanoDrop, Wilmington, DE, USA). Specifically, OD260/OD280 ratios between 1.8 and 2.1 deemed acceptable, while OD260/OD230 ratios of greater than 1.8 deemed acceptable. RNA integrity and DNA contamination were then assessed using electrophoresis on a denaturing agarose gel. In order to enrich pure circRNAs, linear RNAs were removed from the total RNA in each sample after treatment with RNase R (Epicentre, Inc.). The enriched circRNAs in each sample were amplified and fragmented into small pieces using divalent cations under an elevated temperature. RNA-seq library preparation was carried out using the NEBNext® Ultra™ Directional RNA Library Prep Kit for Illumina. Sequencing of the libraries was conducted on an Illumina HiSeq™ 3000 system following the vendor’s recommended protocol (TruSeqR RNA Sample Preparation v2 Guide, Illumina Company Ltd).

HTSeq software was used to count the reads numbers mapped to each circRNA. RPM (Reads Per Million mapped reads, including TopHat mapping and TopHat-Fusion mapping) [1] was employed to calculate the expression level of individual circRNA. Differential expression between circRNAs was assessed by DEGseq algorithm. We defined the statistical criteria for selecting aberrant-expressed circRNA using a q-value < 0.05 with a fold change > 2.0 or < 0.5.

## Quantitation of RNA using quantitative real-time reverse-transcription polymerase chain reaction (qRT-PCR)

Following RNA extraction from the LSCC and matching normal tissues (*n* = 10 pairs), cDNA was synthesized with a reverse transcriptase according to the kit’s instructions (SuperScript First-Strand Synthesis System for RT-PCR, Invitrogen, Carlsbad, CA, USA). circRNA expression was measured using qPCR (SYBR Green PCR Master Mix, Applied Biosystems, Foster City, CA, USA), conducted in a 20-μl reaction volume consisting of the following agents: 6.8 μl cDNA, 10 μl 2 x SYBR Green mix, 0.8 μl primer forward (5 μM), 0.8 μl primer reverse (5 μM), and 1.6 μl H2O. The qPCR reaction was then performed on an ABI 7300 Real-Time PCR System (Applied Biosystems): one 2-min cycle at 50 °C, one 10-min cycle at 95 °C, forty cycles of 15 s at 95 °C and 1 min at 60 °C. β-actin served as a control. PCR reactions were conducted three times. Relative circRNA expression was calculated using the 2 − ΔCt method.

Statistical analyses were performed using GraphPad Prism 5 (GraphPad Software, CA, USA), R software version 3.2.1 (http://www.r-project.org/) and Microsoft Excel (Microsoft, DC, USA). Student’s t-test was used to determine significance for differences in circRNA expression. A two-sided *P* value < 0.05 was considered statistically significant.

## Prediction of circRNA-miRNA interactions

As circular RNAs were shown to have an impact on miRNA-mediated regulation of gene expression through miRNA sequestration [9], putative circRNA/miRNA interactions for the 12 differential circRNAs identified from the microarray and qRT-PCR validation experiments were predicted using the Arraystar miRNA target prediction software (Arraystar), based on TargetScan and miRanda algorithms [9]. The Arraystar software was then used to search for MREs on the 12 differential circRNAs to construct the circRNA-miRNA network [9]. After that, putative miRNAs were identified based on their seed-sequence complementarity using the circRNA-miRNA network. The parameters used for this Arraystar MRE analysis as previously described included [9]: 1) seed type (seed-sequence complementarity) between nucleotides 2–7, 2) proximity of AU-richness to seed sequence, and 3) proximity to nucleotides pairing with the miRNA’s 3′ pairing sequence (nucleotides 13–16). The map of the circRNA/miRNA interaction network was then diagrammed using cytoscape 3.01.

## Prediction of cancer-related circRNA-miRNA-target gene associations

Several online bioinformatics platforms were used in conjunction to predict circRNA-miRNA-target gene associations. First of all, miRNA candidates were identified in KEGG cancer-related pathways within the circRNA-miRNA network using the DIANA-miRPath v.3 platform, which has been reported in previous study [10], the candidate circRNA(s) were matched against the seed sequences of predicted target miRNA candidate(s) using the IntaRNA platform to predict interactions between RNA molecules. The settings that were applied to the DIANA-miRPath v.3 platform included: 0 suboptimal interactions, 5 minimum number of basepairs in seed, 0 maximum number of mismatches in seed, 37.0 temperature for energy computation, 22 folding window size target, and 22 max. Basepair distance (target). Finally, after validation of circRNA-target miRNA interaction, identification of the top ten-ranking cancer-related target genes for each of the target miRNA candidates were then performed using the DIANA-TarBase 7.0 database [10].

## Findings

### Characteristics of the human SLCC samples

Based on the histopathological vetting, a total of ten fresh tissue samples consisting of five LSCC samples (including three moderately differentiated and two well differentiated samples), and five matching adjacent normal tissue samples were finally detected in the study. The detailed information on patients’ characteristics and CONSORT flowchart describing the selection of tested tissue samples was provided in Additional file 1: Figure S1 and Additional file 1: Table S1.

### Identification of differential circRNA profiles

**Fig. 1:**
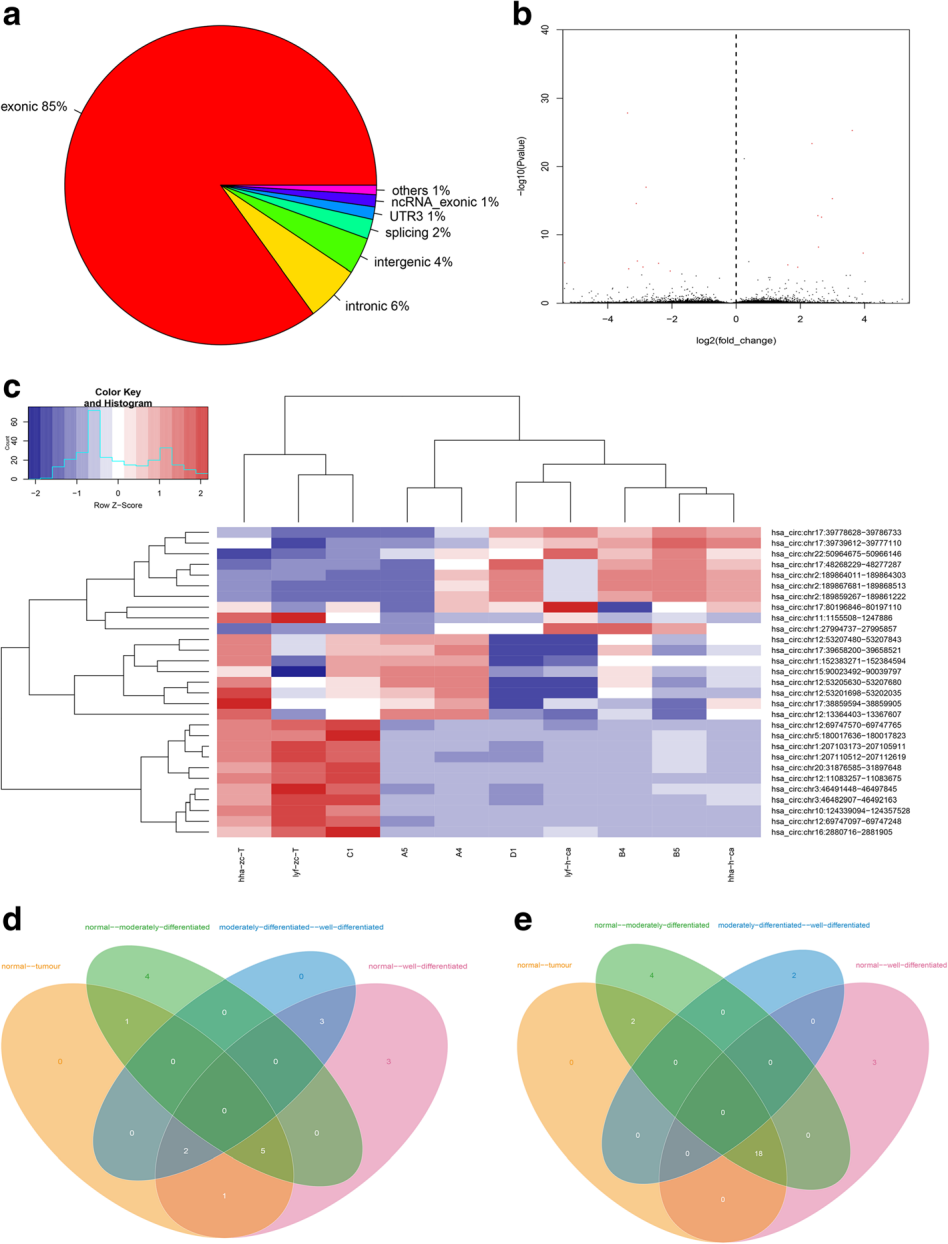
The secondary sequencing information of differential circRNA profiles from the LSCC samples. **a** The percentage of significantly differentially expressed circRNAs arising from different genomic locus (exonic, intronic, antisense, intragenic, and intergenic). **b** Volcano plot. X-axis: the fold change expressed as log2; Y-axis: *P* value expressed as -log10. The vertical green lines corresponded to 2.0-fold up and down, and the horizontal green line represented a *p*-value of 0.05. The red points in the plot represented circRNAs that were expressed differentially with statistical significance. **c** Hierarchical clustering of the circRNA expression data according to ‘All Targets Value’. It classified the samples into different groups based on their expression levels. It revealed a distinguishable circRNA expression profiling among the samples used in this study. **d** & **e** The overlapping significantly changed circRNAs in LSCC versus the normal adjacent tissue. There were 20 significantly downregulated (**d**) and 9 upregulated (**e**) circRNAs in the LSCC versus the normal adjacent tissues (yellow area). There were 24 significantly downregulated (**d**) and 10 upregulated (**e**) circRNAs in moderately differentiated tumors versus the normal adjacent tissues (green area). There were 21 significantly downregulated (**d**) and 15 upregulated (**e**) circRNAs in well differentiated tumors versus the normal adjacent tissues (red area). Integrating these three comparisons, we found 18 overlapping significantly downregulated and 5 upregulated circRNAs in LSCC versus the normal adjacent tissues. These 23 significantly changed circRNAs were detailed in Additional file 1: Table S3

The secondary sequencing was performed to profile circRNA expression in the laryngeal carcinoma samples. The sequencing results showed that 21,444 circRNA were detected, in which about 85% of reads were covered in the exon of genomic (Fig. 1a). After identification of differentially expressed circRNAs by using fold-change filtering (|log2(fold change)| > 1) and Student’s t-testing (Q-value < 0.05) (Fig. 1b), compared to the normal laryngeal tissue, there were 29 circRNAs that were significantly upregulated in LSCC and 19 circRNAs that were significantly downregulated in LSCC (Fig. 1d and e, Additional file 1: Table S2). Hierarchical clustering was then performed to demonstrate the circRNA expression patterns among the samples (Fig. 1c).

### Cluster screening from the different group of the laryngeal carcinoma samples

Calculating the intersection of different expression circRNA from normal-well differentiated set and normal-moderately differentiated set, we were able to narrow candidate genes down to 23. Figure 1d and e was the Venn diagram of this intersection. Among them, 18 significantly downregulated circRNAs were overlapping between the foregoing comparisons, indicating the highest fold-changes (in descending order; Fig. 1d, Additional file 1: Table S3). Meanwhile, 5 significantly upregulated circRNAs that were overlapping between the foregoing comparisons were also identified (in descending order) (Fig. 1e, Additional file 1: Table S3).

### Establishment of the circRNA-miRNA network

**Fig. 2:**
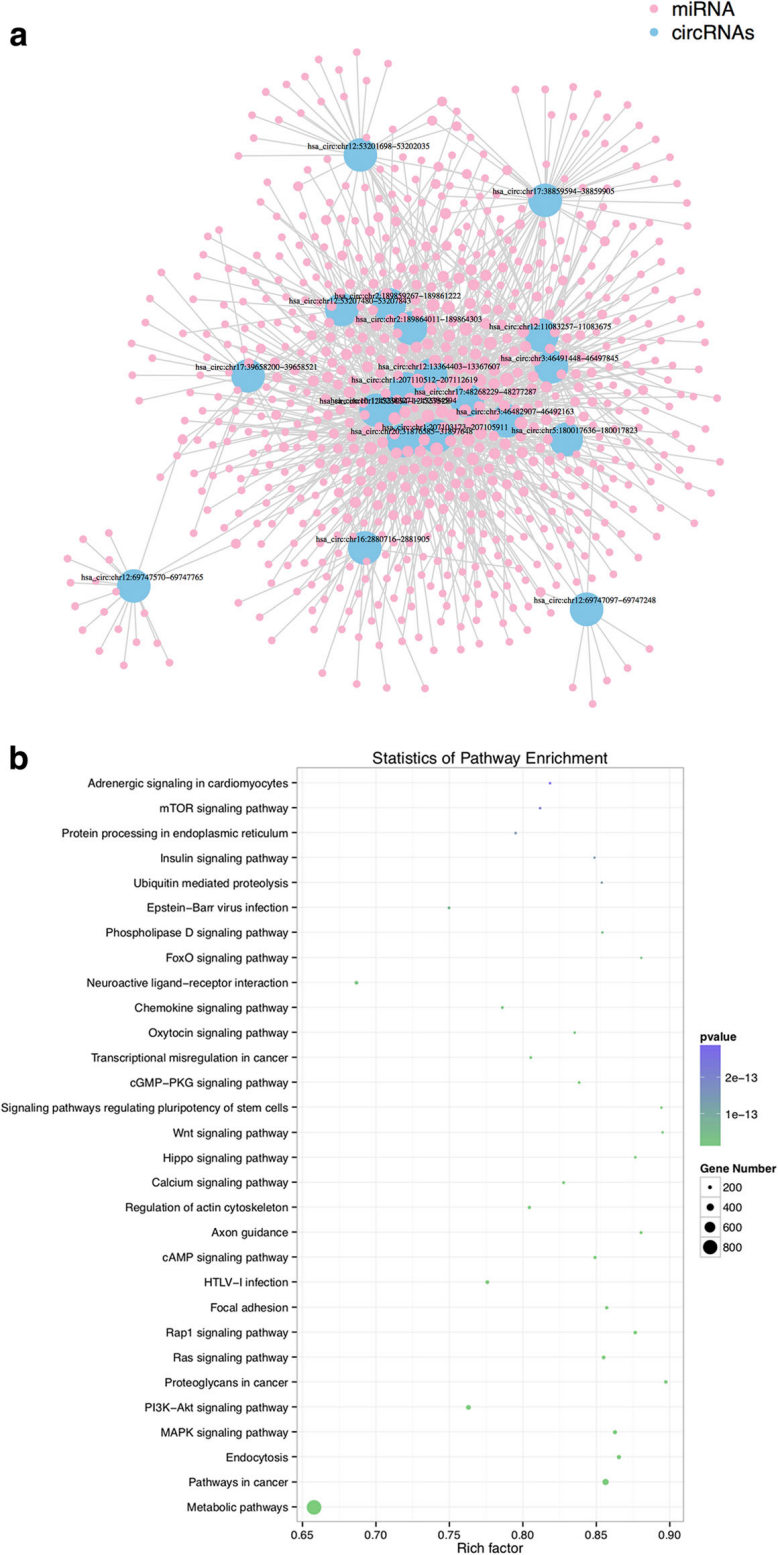
**a** The mapping network of circRNA-miRNA interactions in LSCC. The network map included the identified 20 of 23 significantly changed circRNAs (represented as blue nodes) in the analysis for circRNA-miRNA network prediction, while the other three changed circRNAs did not show reliable results in this prediction. The red nodes around the blue one were the predicted miRNA that were interacted with the related circRNA. The numerical rank of each circRNA fold-change was annotated next to each circRNA node. **b** Bulb map of KEGG analysis for 20 circRNA interacted miRNA and their target gene related significant enriched signaling pathway. X-axis represented the ratio of enriched differential gene in each pathway. Y-axis showed the name of statistics pathway enrichment. The area of each node represented the number of enriched differential genes. The *p*-value were indicated by different color changes from green to purple

Due to interaction of circRNAs with miRNAs via miRNA response elements (MREs), putative MREs were searched for through Arraystar’s circRNA target prediction software. The predicted MREs for the 3 of 5 significantly-upregulated circRNAs were listed in Additional file 1: Table S3. As there were two unreliable predictions in upregulated circRNAs, we were unable to match them to higher frequency of targeting. The predicted MREs for the 17 significantly downregulated circRNAs were also listed in Additional file 1: Table S3. Based on these dysregulated circRNAs and their predicted MREs, a network map of circRNA-miRNA interactions with Cytoscape was established (Fig. 2a).

### Prediction of the characteristics of the circRNA signature related to LSCC using gene ontology and KEGG analyses

Current literature has shown that the circRNA serves as miRNA sponges in some circumstances and regulate gene expression. Based on this, we ranked and identified miRNAs for LSCC related circRNAs. We included the top five ranking candidate miRNAs for each circRNA through specific base pairing. Moreover, we investigated the functions of their target genes using Gene Oncology (GO) and Kyoto Encyclopedia of Genes and Genomes (KEGG) pathway analysis. It demonstrated that the target genes that were related to this circRNA signature participated in various biological processes, such as developmental process, cellular regulation and cell junction (Additional file 1: Figure S2). The altered circRNAs could also have an impact on several vital pathways, such as MAP kinase (MAPK) signaling pathway, Phosphatidylinositol 3-Kinase–AKT (PI3K-Akt) signaling pathway, and Ras signaling pathway (Fig. 2b and Additional file 1: Figure S3).

### Prediction of cancer-related circRNA-miRNA-target gene associations

**Fig. 3:**
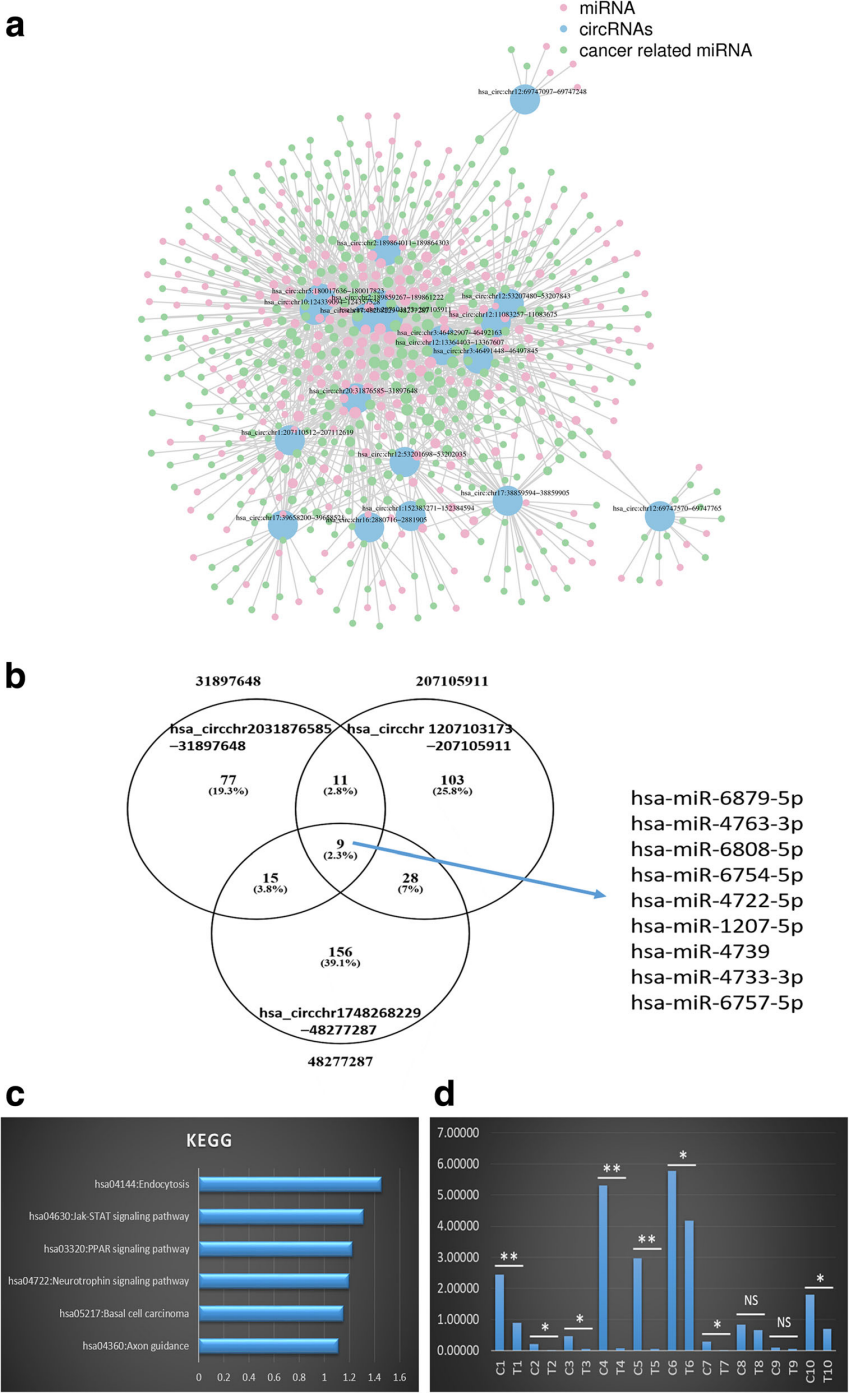
**a** Prediction of circRNA-miRNA associations. The cancer related miRNA was annotated by green nodes, and the number of cancer related miRNA to each circRNA (blue nodes) was counted in Additional file 1: Table S3. The common putative signaling pathway analysis for three candidate circRNAs were shown as below. **b** The venn map showed the nine common miRNAs regulated by three candidate cancer related circRNAs (hsa_circ:chr17:48268229–48,277,287, hsa_circ:chr 1:207103173–207,105,911 and hsa_circ:chr20:31876585–31,897,648). These nine miRNAs were shown in the right side of the venn map. **c** The putative signaling pathway was analyzed by KEGG using the targeted genes of these nine common miRNAs. **d** Downregulation of hsa_circ:chr20:31876585–31,897,648 in the LSCC tissues identified by Q-PCR. Ten paired LSCC tumor (T1-T10) and their adjunct tissues (C1-C10) were used to test the relative expression level of the candidate circRNA of hsa_circ:chr20:31876585–31,897,648. It was downregulated in eight LSCC samples compared with their adjunctive normal tissues (No Significant, NS; *p* < 0.05, *; *p* < 0.01, **)

To better understand the underlying molecular mechanism(s) of the differential circRNAs, we first selected the miRNA from KEGG analysis that enriched in pathway in cancer (Fig. 2b, Additional file 1: Figure S3) and used the bioinformatics platform to profile the cancer-related miRNA network. Then, the predicted cancer-related circRNA-miRNA associations were showed in Fig. 3a. Additional file 1: Table S3 indicated the number of each dysregulated circRNA that were interacted with cancer-related miRNA was annotated in Fig. 3a (green nodes). Finally, we selected the top three circRNA (hsa_circ:chr17:48268229–48,277,287, hsa_circ:chr20:31876585–31,897,648, hsa_circ:chr 1:207103173–207,105,911) which could bind to 151, 112 and 208 cancer related miRNA, respectively (Additional file 1: Table S3). By comparing and overlapping the lists of these three top-ranking cancer-related circRNAs, several promising cancer-related pathways were able to be identified, which may be potential targets of these circRNA/miRNA/ mRNA axis in LSCC (Fig. 3a).

### Top three candidate circRNA that were involved in the main regulation of the PPAR, AXON-guidance, Wnt and cell cycle signaling pathway in LSCC development

Three candidate circRNAs (207,105,911, 31,897,648 and 48,277,287) were selected, and their corresponding numbers of cancer related miRNAs were 151, 112 and 208, respectively. Interestingly, nine common targeted cancer related miRNAs were found in these three candidate circRNAs (Fig. 3b). Further KEGG analysis were performed using these nine miRNA-target genes (Fig. 3c), which elucidated that PPAR, Axon guidance, Wnt and Cell cycle signaling pathway were found to be a key putative signaling pathway in the process of LSCC.

At last, three candidate circRNAs were prepared for validation. As no specific primer can be designed for detecting hsa_circ:chr17:48268229–48,277,287 and hsa_circ:chr 1:207103173–207,105,911, only hsa_circ:chr20:31876585–31,897,648 was selected for qRT-PCR validation. T1 (the tumor tissues) and C1 (control, the adjunct normal tissues) were a paired sample from the first patient. All paired samples from ten patients also showed the downregulation of hsa_circ:chr20:31876585–31,897,648 in the LSCC tissues (T1-T10) compared to each of their adjunct tissues (C1-C10) (Fig. 3d). It demonstrated that hsa_circ:chr20:31876585–31,897,648 was significantly differentiated between most of the ten paired LSCC tumor tissues and the matching normal tissues. It indicated that circRNA hsa_circ:chr20:31876585–31,897,648 may have the potential to be a novel biomarker for LSCC in future research.

In this study, we have profiled the circRNA expression in LSCC in details using secondary sequencing to improve our understanding of the pathogenesis of LSCC. We have also explored a potential role of this novel circRNA biomarker used for the diagnostic and prognostic value of the disease. From sequencing and subsequent overlapping analysis, a total of 20 significantly candidate differentiated circRNAs in LSCC were identified, including 16 upregulated and four downregulated, compared to the adjacent normal tissues. Four significantly upregulated circRNAs were identified as the most potential biomarkers related in LSCC.

Furthermore, a network map of circRNA-miRNA interactions was also established for total 20 significantly differentiated circRNAs using a set of bioinformatics tools. The functions of their target genes were then assessed with the Gene Oncology (GO) analysis. It demonstrated that the target genes that were related to these 20 dysregulated circRNA participated in various biological processes, such as growth factors binding, protein phosphatase activator activity, skin development, wound healing, most of which were related to proliferation process. Moreover, Kyoto Encyclopedia of Genes and Genomes (KEGG) pathway analysis was performed to manifest some vital pathways, including Ras, MAPK, PI3K-AKT cancer related pathways, involved in the altered circRNAs in the LSCC samples.

Previous molecular research on LSCC has primarily focused on genomic analyses. In recent years, researchers have also assessed changes in the circRNA expression in LSCC via microarray [7]. However, as the relationship of circRNA-miRNA-mRNA-based research is an emerging area of investigation [10]. to our knowledge, there is no study to date has yet assessed circRNA in LSCC based on these relationships. Therefore, our study comprehensively profiled the relationship of 20 candidate circRNAs and their possible interactions with miRNAs. Based on a hypnosis that the more important circRNAs could interact with more different cancer related miRNAs and further regulated different cancer related pathways. Hence the top three dysregulated circRNAs - that have been predicted could bind nine common cancer related miRNAs in LSCC - were further validated by qRT-PCR. Then the common miRNA targeted 576 mRNA were analyzed by KEGG. The KEGG annotation indicated that these three circRNA may mainly regulate (involve in) the PPAR, Axon guidance, Wnt and Cell cycle signaling pathway. Therefore, we presumed that these pathways may be potential therapeutic target in LSCC.

Our study has several limitations. First of all, as a single centre study, it has a relatively small sample size, which has an impact on the statistical power. Further larger studies are needed to assess the role of circRNAs in LSCC. Secondly, the association of circRNAs and severity of LSCC was unable to be assessed in this study due to the limited number of LSCC samples, which also warrants further investigations.

## Conclusions

In conclusion, the high-throughput transcriptome sequencing and bioinformatics analysis of the laryngeal squamous cell carcinoma provides useful diagnostic markers and potential therapeutic targets for the future research of this disease.

## Additional file

Additional file 1: **Table S1.** Demographic characteristics of patients with LSCC involved in this study. **Table S2.** Individual circRNAs detected in the study (please see the attached excel spreadsheet). **Table S3.** LSCC specific circRNAs detected in the study (please see the attached excel spreadsheet). **Figure S1.** The representative images of H&E-stained normal laryngeal mucosal and LSCC specimens. Pictures included well differentiated (upper), moderately differentiated LSCC (middle), and normal tissues (lower). From left to right, image magnifications of 40×, 100×, 200×, and 400 × were displayed (scale bar = 100 μm). **Figure S2.** Gene Ontology annotation analysis for 20 circRNA interacted miRNA and their target gene related significant enriched biological process, cellular components and molecular function. **Figure S3.** KEGG analysis showing the map of pathway in LSCC using the dysregulated circRNA-miRNA-target genes. (ZIP 8463 kb)

## Abbreviations

circRNAs: Circular RNAs
GO: Gene Oncology
KEGG: Kyoto Encyclopedia of Genes and Genomes
LSCC: Laryngeal squamous cell carcinomas
MAPK: MAP kinase
PI3K-Akt: Phosphatidylinositol 3-Kinase–Akt
