# Supplementary material for: RNA-Seq profiling of circular RNAs in human laryngeal squamous cell carcinomas
Source: Mol Cancer. 2018 May 1;17:86. doi: 10.1186/s12943-018-0833-x (PMC5930968; doi:10.1186/s12943-018-0833-x)

**RNA-Seq profiling of circular RNAs in human laryngeal squamous cell carcinomas**

**Supplementary materials**

Table S1： Demographic characteristics of patients with LSCC involved in this study

| Group | Sample ID | Age (yrs) | Sex | Previous personal history of LSCC | Family history of LSCC |
| --- | --- | --- | --- | --- | --- |
| LSCC tissues | B4 | 46.5 | M | None | None |
| Adjacent non-tumor tissues(normalized) | A4 | 46.5 | M | None | None |
| LSCC tissues | B5 | 58.1 | M | None | None |
| Adjacent non-tumor tissues(normalized) | A5 | 58.1 | M | None | None |
| LSCC tissues | D1 | 64.7 | M | None | None |
| Adjacent non-tumor tissues(normalized) | C1 | 64.7 | M | None | None |
| LSCC tissues | Hha-h-ca | 67.0 | M | None | None |
| Adjacent non-tumor tissues(normalized) | Hha-zc-T | 67.0 | M | None | None |
| LSCC tissues | LYf-h-ca | 81.8 | M | None | None |
| Adjacent non-tumor tissues(normalized) | LYf-zc-T | 81.8 | M | None | None |

Table S2： Individual circRNAs detected in the study (please see the attached excel spreadsheet)

Table S3： LSCC specific circRNAs detected in the study (please see the attached excel spreadsheet)

Figure S1. The representative images of H&E-stained normal laryngeal mucosal and LSCC specimens. Pictures included well differentiated (upper), moderately differentiated LSCC (middle), and normal tissues (lower). From left to right, image magnifications of 40×, 100×, 200×, and 400×were displayed (scale bar=100μm)


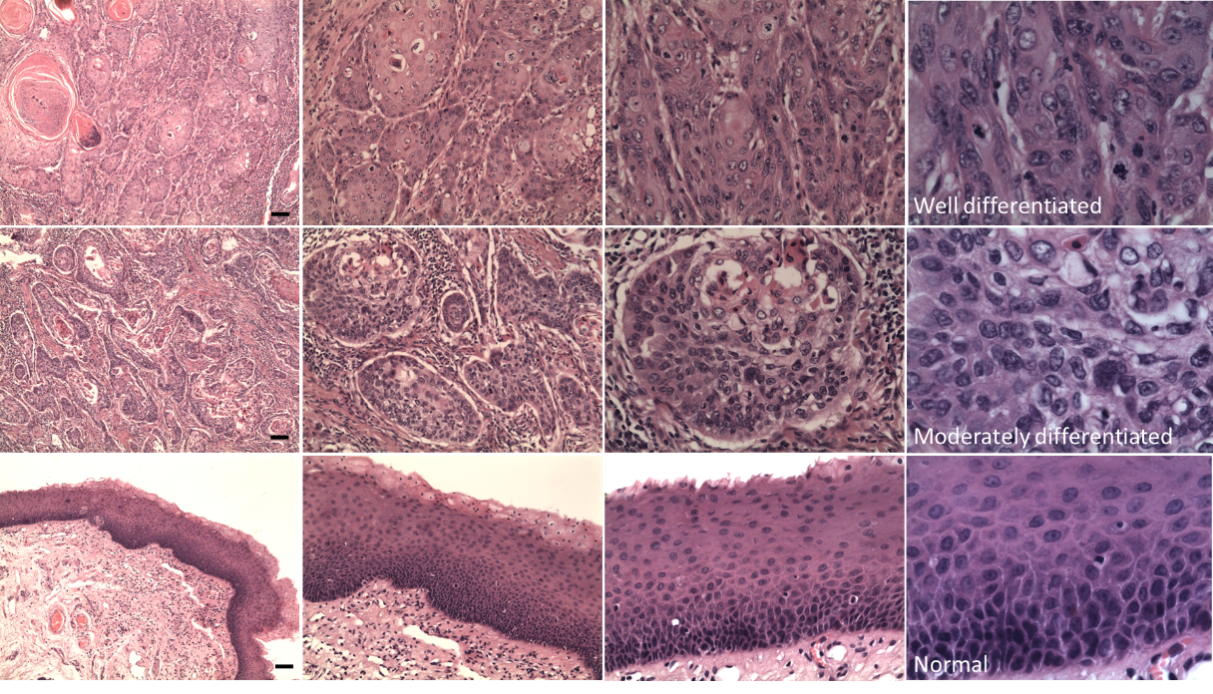


Figure S2 Gene Ontology annotation analysis for 20 circRNA interacted miRNA and their target gene related significant enriched biological process, cellular components and molecular function.


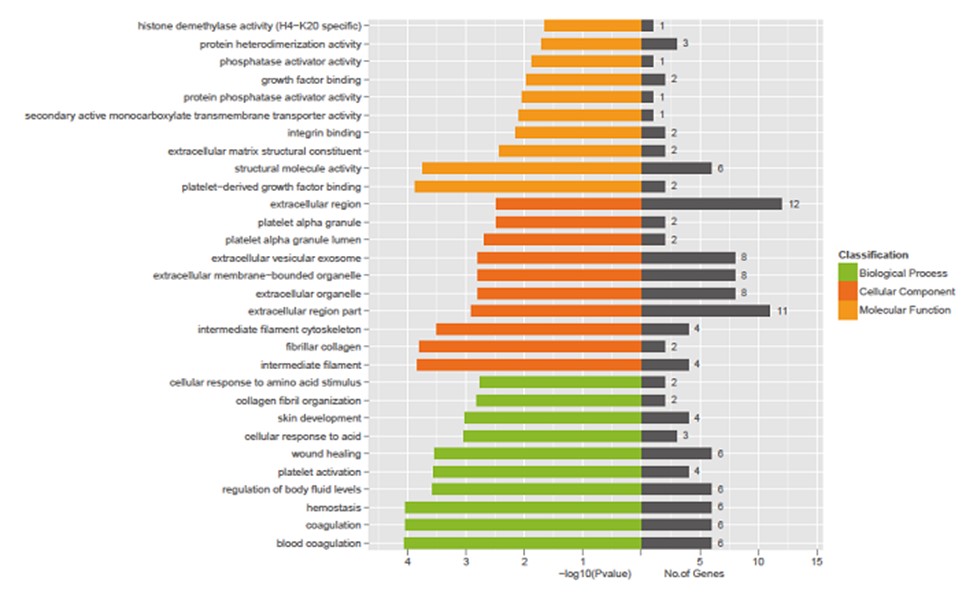


Figure S3 KEGG analysis showing the map of pathway in LSCC using the dysregulated circRNA-miRNA-target genes.


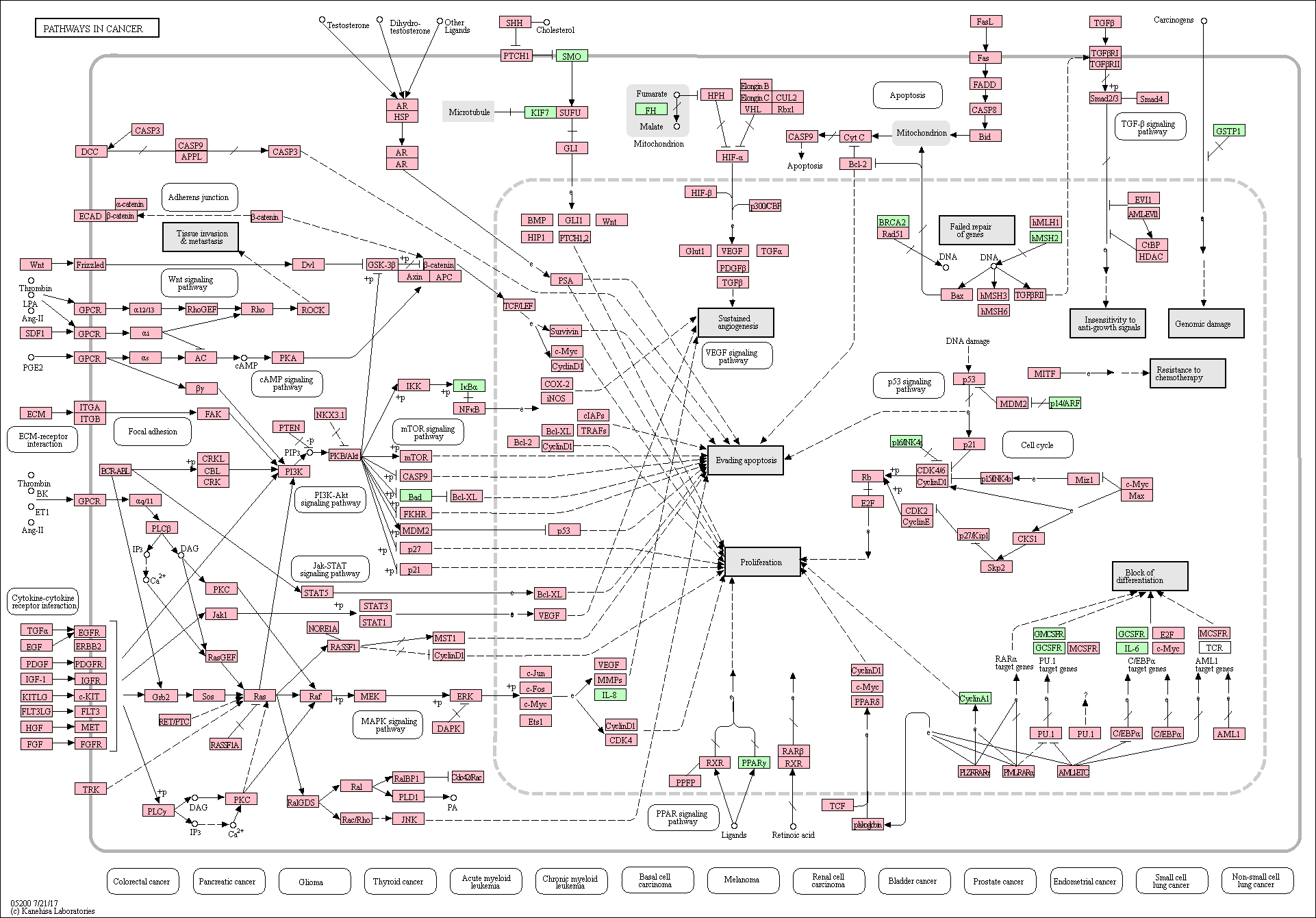

Supplement: Supplementary file 1 — Table S1. Demographic characteristics of patients with LSCC involved in this study. Table S2. Individual circRNAs detected in the study (please see the attached excel spreadsheet). Table S3. LSCC specific circRNAs detected in the study (please see the attached excel spreadsheet). Figure S1. The representative images of H&E-stained normal laryngeal mucosal and LSCC specimens. Pictures included well differentiated (upper), moderately differentiated LSCC (middle), and normal tissues (lower). From left to right, image magnifications of 40×, 100×, 200×, and 400 × were displayed (scale bar = 100 μm). Figure S2. Gene Ontology annotation analysis for 20 circRNA interacted miRNA and their target gene related significant enriched biological process, cellular components and molecular function. Figure S3. KEGG analysis showing the map of pathway in LSCC using the dysregulated circRNA-miRNA-target genes. (ZIP 8463 kb) [file 12943_2018_833_MOESM1_ESM.zip › cRNA_supplementary materials_aw4.6.docx]
